# Response of human normal and leukemia cells to factors released by amnion fragments *in vitro*

**DOI:** 10.1371/journal.pone.0195035

**Published:** 2018-03-29

**Authors:** Zofia Grzywocz, Grazyna Hoser, Stanislawa Sabalinska, Piotr Ladyzynski, Jaroslaw Czubak, Malgorzata Dworczynska, Romuald Debski, Ewa Pius-Sadowska, Boguslaw Machalinski, Jerzy Kawiak

**Affiliations:** 1 Department of Clinical Cytophysiology, Medical Centre of Postgraduate Education, Warsaw/Poland; 2 Laboratory of Flow Cytometry, Medical Centre of Postgraduate Education, Warsaw/Poland; 3 Nalecz Institute of Biocybernetics and Biomedical Engineering PAS, Warsaw/Poland; 4 Clinic of Pediatric Orthopedy & Traumatology, Medical Centre of Postgraduate Education, Warsaw/Poland; 5 Obstetrics and Gynecology Clinic, Bielanski Hospital, Warsaw/Poland; 6 Department of General Pathology, Pomeranian Medical University, Szczecin/Poland; Centro Cardiologico Monzino, ITALY

## Abstract

Amnion is a membrane surrounding the embryo/fetus which determine growth factors and interleukins with angiogenic, immunogenic, and anti-inflammatory properties. The aim of the present study was to investigate the effects of conditioned culture medium from 24-h cultures of human amnion (hAM CCM) on migration and proliferation of human umbilical vein endothelial primary cells (HUVECs), freshly isolated bone marrow mononuclear cells (BM MNCs), and Jurkat leukemia cell line. Amnion membrane was freshly isolated from healthy placenta and its fragments cultured *in vitro* to produce hAM CCM. Members of the IGFBP protein family made up one third of all assayed proteins present in the hAM medium. The hAM CCM did not affect the proliferation rate of HUVECs or MNCs, but we observed more intensive migration of those cells, and lower expression of CD31 surface antigen on HUVECs as compared to control cultures. In contrast, Jurkat cells did not respond to hAM CCM treatment by proliferation or mobility change. The conditioned medium from 24-h cultures of human amnion is easy to obtain and is a convenient source of various growth and other factors that may be useful in practical medicine.

## Introduction

Amnion is a thin membrane surrounding the embryo/fetus, without a supply of blood vessels. Two populations of cells can be distinguished within the human amnion: epithelial cells and mesenchymal stromal/stem cells. The amniotic membrane (AM) functions are angiogenic, immunogenic, and anti-inflammatory [1]. Amniotic membrane is a convenient source of cells producing potentially therapeutic factors [2]. A successful use of AM has been described, for example, in the treatment of venous leg ulcers [3], promotion of wound healing by facilitating migration and proliferation of keratinocytes [4], in dermatology and ophthalmology [5, 6, 7], especially in caustic burns of the eye [8]. The beneficial properties of AM transplants arise from their bacteriostatic properties, pain reduction, epithelialization effects, and an absence of induction of immunological response [3,7,8]. It seems therefore rational to test *in vitro* the effects of the components released by AM on cell mobility and proliferation to find the basis of the observed positive therapeutic effects.

The aim of the present study was to obtain conditioned culture medium of human amnion (hAM CCM) and to test its effect on the migration and proliferation properties of normal human cells active in the healing process: human umbilical cord endothelial cells (HUVECs) and human progenitor and bone marrow mononuclear cells (BM MNCs). The response of the target normal cells tested was compared with the responses of human leukemia Jurkat cells.

## Materials and methods

### Biological material

Human AMs and HUVECs were freshly isolated from placentas and umbilical cords of 18 healthy women from the Second Obstetrics and Gynecology Clinic of Bielanski Hospital, Warsaw, Poland. Bone marrow was obtained from patients with pelvic osteotomy from the Clinic of Pediatric Orthopedy & Traumatology, Medical Centre of Postgraduate Education, Warsaw. Study protocols were approved by the Bioethical Commission at the Medical Centre of Postgraduate Education (decisions of 05.12.2012 and 23.03.2015), and informed consent was obtained from all subjects. Materials from patients were collected between 2012 and 2017. Human Jurkat immortalized T cell line was obtained from the American Type Culture Collection (ATCC, CRL-2063).

### Amnion fragments and preparation of conditioned culture medium

Amnion fragments were prepared according to a protocol described previously [9], with minor modifications. Amnions were mechanically separated from placentas and cut into pieces. These fragments were washed twice in phosphate-buffered saline (PBS, pH = 7.4, Sigma-Aldrich, St. Louis, MO, USA) and incubated for 30 min in PBS with penicillin (7.5 × 10^5^ IU/mL, Polfa Tarchomin, Poland), streptomycin (250 mg/mL, Polfa Tarchomin), and nystatin (2.5 mg/mL, Sigma-Aldrich). After another wash with PBS, the amniotic fragments were cut with a Biopsy Punch (Miltex, York, PA, USA) to obtain 5-mm diameter circles which were then placed in a 48-well culture plate (10 circles per well). The amniotic circles were incubated in RPMI 1640 medium with standard concentration of penicillin (1.5 IU/ml) and streptomycin (5 mg/mL) (SPS) for 24 h at 37^°^C, in an atmosphere of 5% CO_2._ The conditioned medium (hAM CCM) was then collected, centrifuged (10 min at 1000 ×*g*), and the supernatant was aliquoted and frozen at –80°C until further use. The sterile hAM CCM was stored in the tissue bank no longer than 3 months for the activity tests.

### Isolation of HUVECs

Umbilical cords were rinsed with PBS and veins were washed with Hank’s balanced salt solution (HBSS, Sigma-Aldrich). Next, umbilical veins were filled with dispase (0.96 mg/mL, Invitrogen Corp., UK) in HBSS and incubated for 15 min at 37°C in a water bath. The liberated cells were collected and centrifuged down at 230 × g. Then, the umbilical veins were washed with minimum essential medium, Medium 199 (Sigma-Aldrich), to retrieve remaining cells, and the cells were collected as above. The cells were pooled and cultured for 4–5 h in Medium 199, the culture medium was changed to Medium 199 supplemented with heat-inactivated 20% fetal bovine serum (FBS, Invitrogen Corp.,UK), heparin (5000 U/ml, Polfa), human endothelial cell growth factor supplement (1 μg/mL, Sigma-Aldrich) with standard SPS and culturing was continued for two weeks. To experimental cultures hAM CCM was added to 10% final concentration (v/v), or RPMI 1640 medium to controls.

### Human bone marrow cells

Bone marrow was washed out during surgical pelvic osteotomy performed for curative purposes. Mononuclear cells were isolated by density gradient centrifugation in SepMate-50 tubes (Stemcell Technologies) filled with 15 mL of Histopaque-1077 (Sigma) and overlayered with the bone marrow suspended in HBSS at 1:1 proportion. The tubes were centrifuged for 20 min at 230 × g, and the separated cells were washed twice with HBSS and counted.

### Jurkat cell line

The cells (ATCC CRL-2063) were cultured in RPMI 1640 medium with 3% heat-inactivated FBS and 2% human albumin (SIS Biomed, Poland) with standard SPS. The culture medium of test cultures was supplemented with hAM CCM to a final concentration of 10%.

### Cytometric analysis

The cell proliferation was probed with an antibody against the marker Ki67 (anti-Ki67 PE, clone B56, BD) with proper PE isotype control (clone B56, BD). Bone marrow MNCs and Jurkat cells were additionally tested to another proliferation marker, proliferating cell nuclear antigen (PCNA) with the PE mouse anti-human PCNA set (clone PC10, BD) with proper PE isotype control (PE mouse Ig2a, kappa, BD). HUVECs were probed for adhesion marker CD31 with PE mouse anti-human CD31 (clone WM59, BD) and PE isotype mouse IgG1 kappa control (clone MOPC-21, BD). All labeled cells were analyzed by flow cytometry with the FACS Canto II cytometer (BD) and Facs Diva software (BD).

### Cytokine antibody array

Tests were performed as described previously [9]. Briefly, we used human antibody array (RayBiotech, Atlanta, GA, USA), which can detect 41 growth factors and binding proteins. The assay was performed according to the manufacturer’s protocol. The array was visualized with a chemiluminescence imaging system (UVP camera GelDocDC Imaging System, BioRad, CA USA), and densitometry analysis was performed with free software: ImageJ with plugin the Protein Array Analyzer (NIH, Bethesda, MD, USA). Fluorescence values (FV) were measured and calculated as follows:

FV = fluorescence of growth factor–MNC/MPC, where MNC–mean of negative control (4 samples), MPC–mean of positive control (6 samples). The FV was assumed the measure of concentration the growth factor.

### Bone marrow MNCs and Jurkat cells migration assays

Migration assays for non-adherent cells were performed in a 24-well Boyden chamber with 8-μm pore size polycarbonate membranes (Corning). Bone marrow MNCs were suspended in GlutaMAX medium with 5% FBS (Life Technologies/Gibco, CA, USA) at 1 x 10^6^ cells/ml. The cell suspension (100 μl) was placed in the upper chamber and the lower chamber was filled with the culture medium supplemented with hAM CCM to 10% or with culture medium alone in controls. The chambers were incubated for 2.5 h at 37^°^C. Thereafter the cells which migrated to the lower chamber were transferred to a test tube, centrifuged, suspended in culture medium and counted using Burker chamber and light microscopy at 100x magnification. The chemotaxy index (CI) was calculated by dividing the number of lower chamber cells by the number of cells added to the upper chamber at the start of the test.

The test for Jurkat cells was performed with minimal modifications. The polycarbonate membranes were first covered with collagen I (Sigma), and the upper well was filled with 2.15 × 10^6^ Jurkat cells in 100 μL of RPMI 1640 with 3% FBS and 2% human albumin. The incubation was for 4 h at 37^°^C.

### HUVEC proliferation and migration assays

The wound healing (scratch) test was applied to assess migration of adherent cells. HUVECs were placed in a fibronectin (Sigma)-covered 12-well dish and cultured for 3 days in Medium 199, as described above. After cell cultures achieved confluence, a 3-mm scratch with a sterile pippete tip was made on the bottom of each well to create a cell-free region. Then hAM CCM was added (to 10%, v/v) to half of the wells and to the other half control medium. Cell growth into the clear area was assessed under an inverted microscope (IX 71, Olympus, Tokyo, Japan) following a 24h incubation at 37^°^C and the cell-free area was quantified using the cellSence Dimension software (Olympus), which is measuring distance between cells migrating to the clear area.

HUVECs were also assayed for migration and proliferation in a real-time system, X-Celligence (Roche, Germany). The X-Celligence system is based on monitoring impedance changes which occur during adhesion and proliferation of cells or are caused by migrating cells seeded in culture chambers with sensors in each well. In the proliferation assay, after determining initial impedance, 6 × 10^3^ HUVECs were added with or without hAM CCM (10%), and for the migration assay, 12 × 10^3^ cells.

### Statistical analysis

The migration and proliferation results were expressed as medians and interquartile ranges (P25–P75) or as unmodified graphs from X-Celligence software. The results of a peptide micro array format (see [9]) were grouped and compared with the Mann–Whitney U-test. The ANOVA test was applied to compare differences among groups of growth factors tested. Post-hoc Tukey’s test and analysis of variance were applied to compare concentration of growth factors. Statistical significance was set at *p* < 0.05. The SAS 9.4 software (SAS Institute Inc, USA) was used.

## Results

### Growth factors released by amniotic membrane

**Fig 1:**
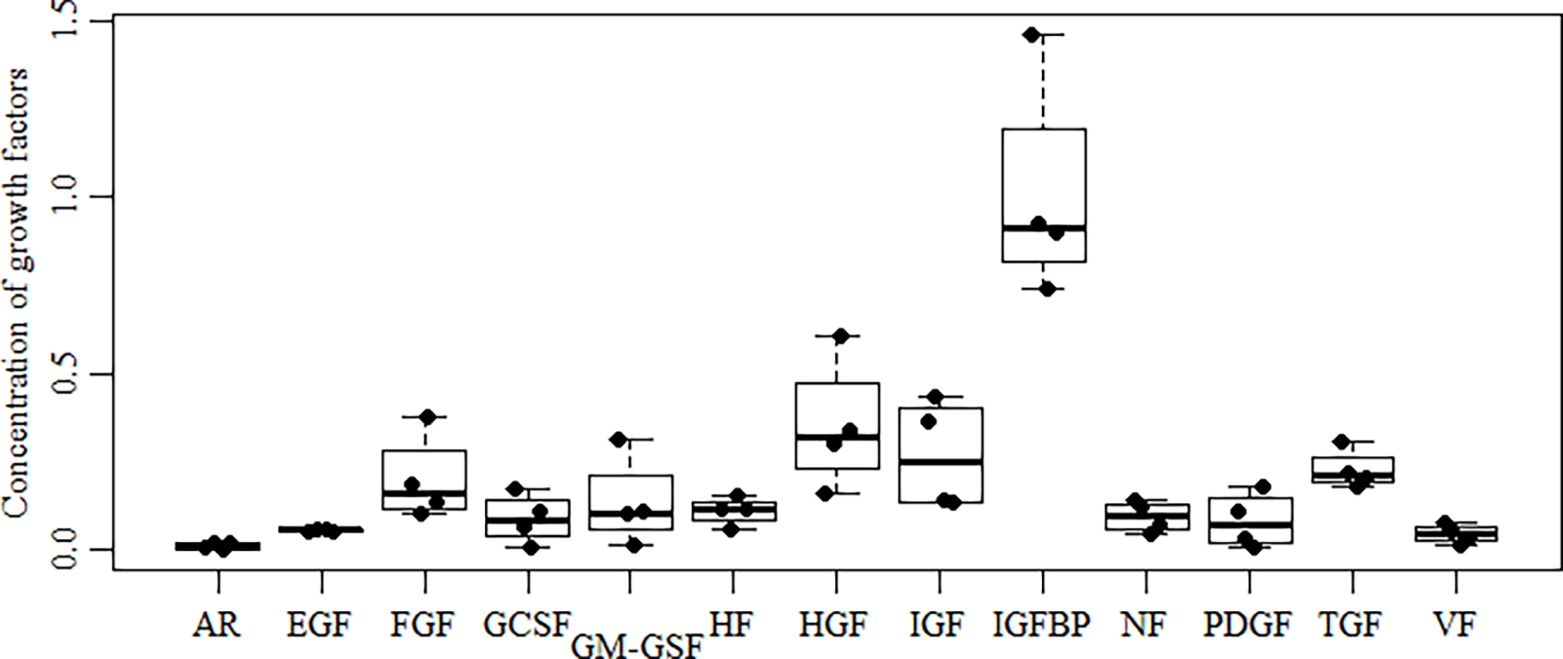
Growth factors in hAM CCM. Forty-one growth factors were quantitated by antibody array and the obtained values were combined into following growth factor families: EGF family (EGF-2, HB-EGF, EGF-R); FGF family (bFGF, FGF-4, FGF-6, FGF-7); Hematopoietic factors HF (MCSF, MCSF-R, SCF, SCF-R); IGF family (IGF-1, IGF-2, IGF-1SR); IGFBP family (IGFBP-1, IGFBP-2, IGFBP-3, IGFBP-4, IGFBP-6); Neurotrophic factors NF (bNGF, GDNF, NT-3, NT-4); PDGF family (PDGF-AA, PDGF-AB, PDGF-BB, PDGF-Ra, PDGF-Rb); TGF family (TGF-α, TGF-β, TGF-β2, TGF-β3); Vasculogenic factors VF (PLGF, VEGF, VEGF-R3, VEGF-D, VEGF-R2); some growth factors are presented separately: AR; G-CSF; GM-CSF; HGF. Each growth factor fluorescence value (FV) was measured and calculated as described in Materials and methods. (n = 4) (p < 0.05).

For preliminary characterization of the content of biologically active factors secreted by the amnion into culture medium (hAM CCM) we used the peptide micro array technique with panel of antibodies directed against human growths factors representing major families: epidermal and fibroblast growth factors (EGF, FGF), hepatocyte growth factor (HGF), vasculogenic and angiogenic growth factors (VEGF, PLGF, AR), neurotrophic factors (NGF, GDNF, NT), hematopoietic growth factors (G-CSF, GM-CSF, M-SCF, SCF), insulin-like growth factors (IGF, IGFBP), platelet-derived growth factors (PDGF), and transforming growth factors (TGF). The quantitations were performed individually for each batch hAM CCM obtained from a single donor. These values were then combined and medians with interquartile ranges are shown in Fig 1. Notably, IGFBPs were three times more abundant than their cofactors, the IGFs. The HGF protein was highly abundant as well.

Having established that numerous growth factors are present in hAM CCM we investigated its effect on migration and proliferation of human normal (HUVECs, BM MNCs) and leukemia cells. The migration assay methods are different for adhering and non-adhering cells, therefore hAM CCM effects is presented separately for different target cells.

### Effect of hAM CCM on proliferation and migration of HUVECs

Proliferation of HUVECs was assayed by measuring the level of Ki67, 24 h after addition of the hAM CCM and independently using proliferation real time X-Celligence system for up to 100 h (S1 Fig). The proliferation rate of HUVECs was very low and neither assay showed a statistically significant effect of the hAM CCM treatment.

**Fig 2:**
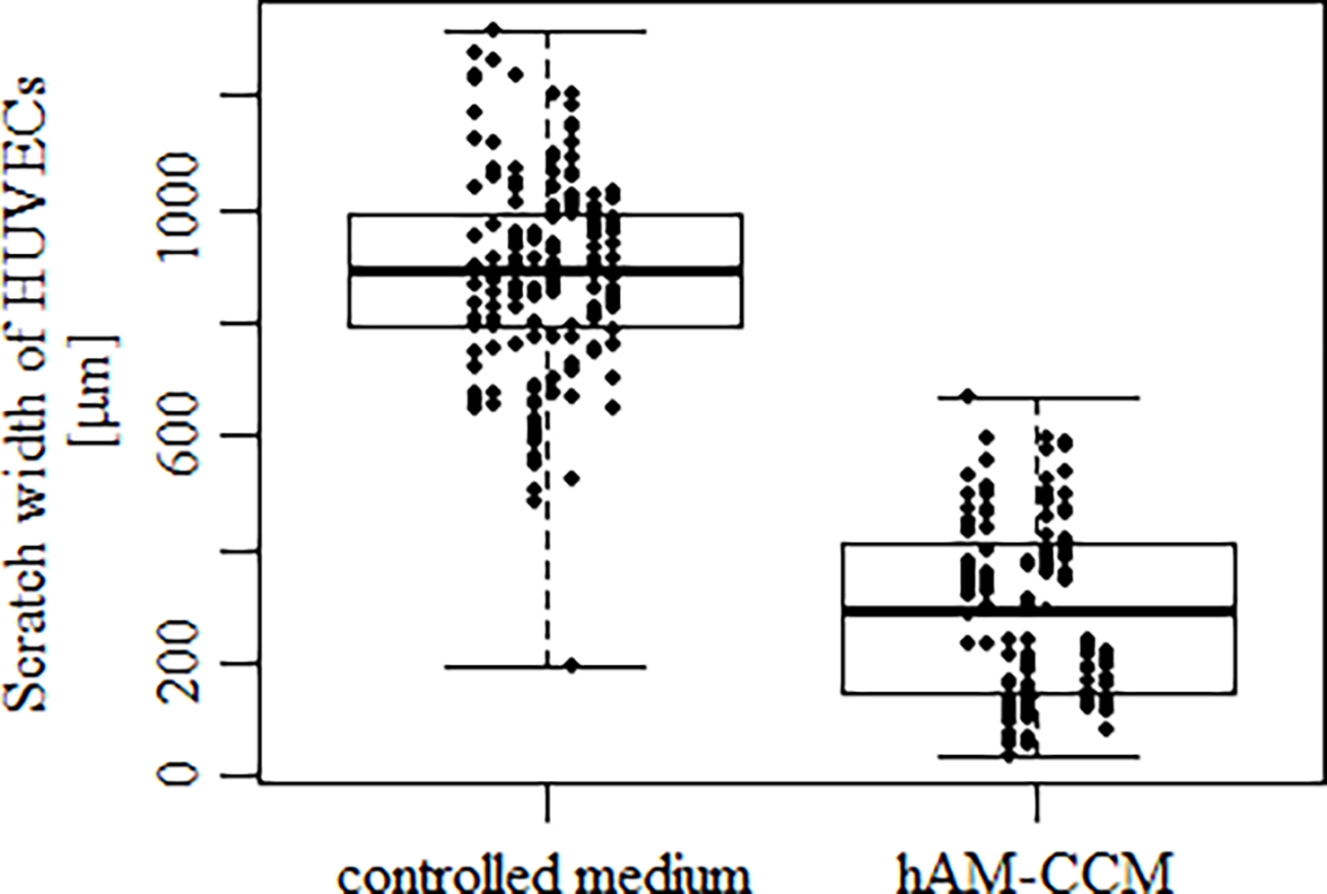
Effect of hAM CCM on HUVECs migration assayed by scratch test. There are results of 160 measurements, 8 independent assays with 10 measurements for test and control each. Median values and (P25, P75) are shown (n = 8, p < 0.05). Detailed description of the assay is in Material and methods.

Migration of HUVECs was tested in the scratch test. The width of the scratch after 24 h in the presence of hAM CCM was significantly smaller then in controls (Fig 2) suggesting faster HUVEC migration in the presence of hAM CCM.

**Fig 3:**
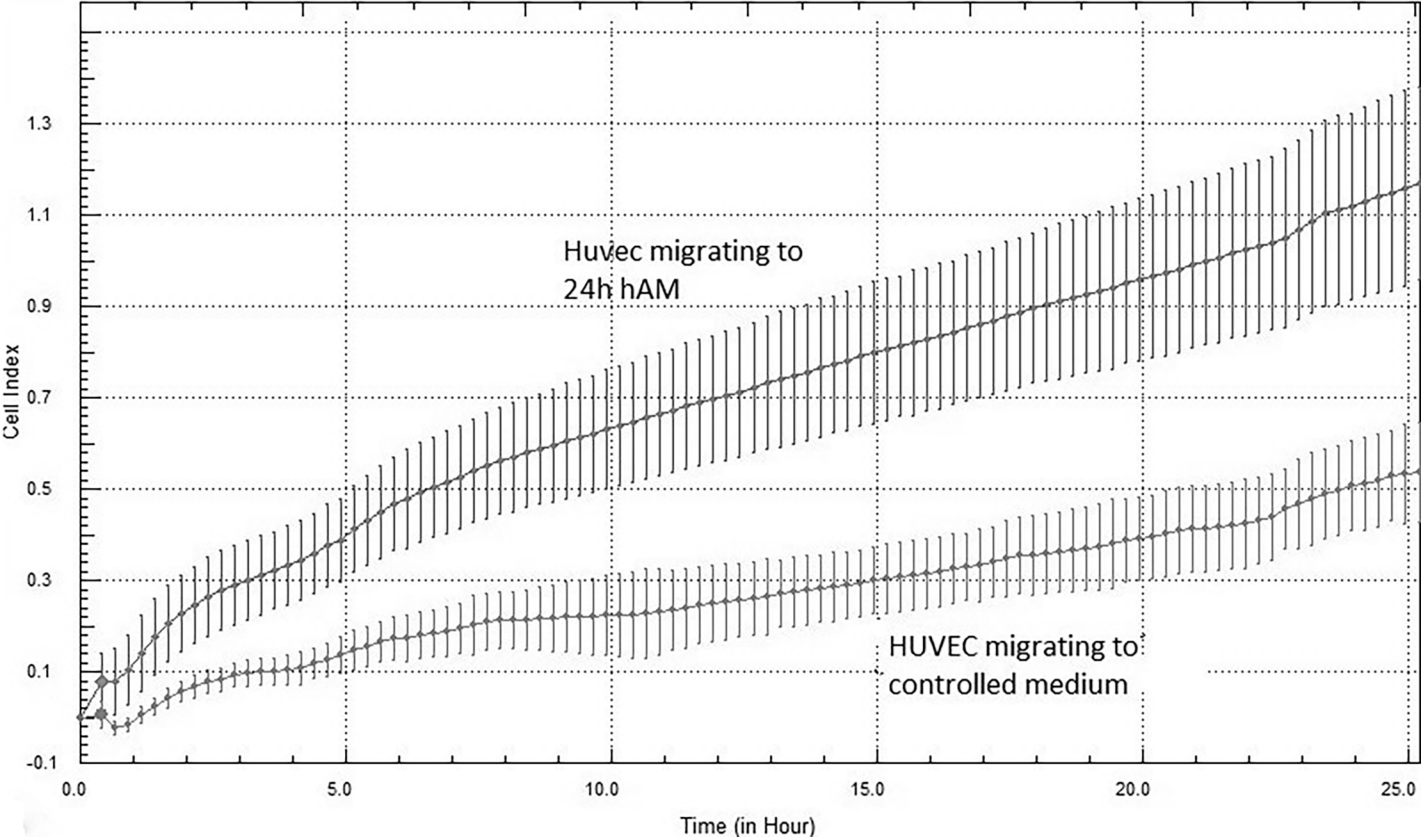
The example of real time migration assay of stimulated and controlled HUVECs directly from the X-Celligence system. The assay was repeated seven times with similar result during 25h observation at 37^°^C. The difference between migration curves for cells in cultures with presence of hAM CCM and in control medium was significant. (p < 0.05).

The difference in migration between experimental and control HUVECs cultures was measured by migration real-time system, X-Celligence (Fig 3). From these results, we may infer that e.g. at 10 h from the addition of hAM CCM, the stimulated adherent cells migrated about three times faster than control cells.

**Fig 4:**
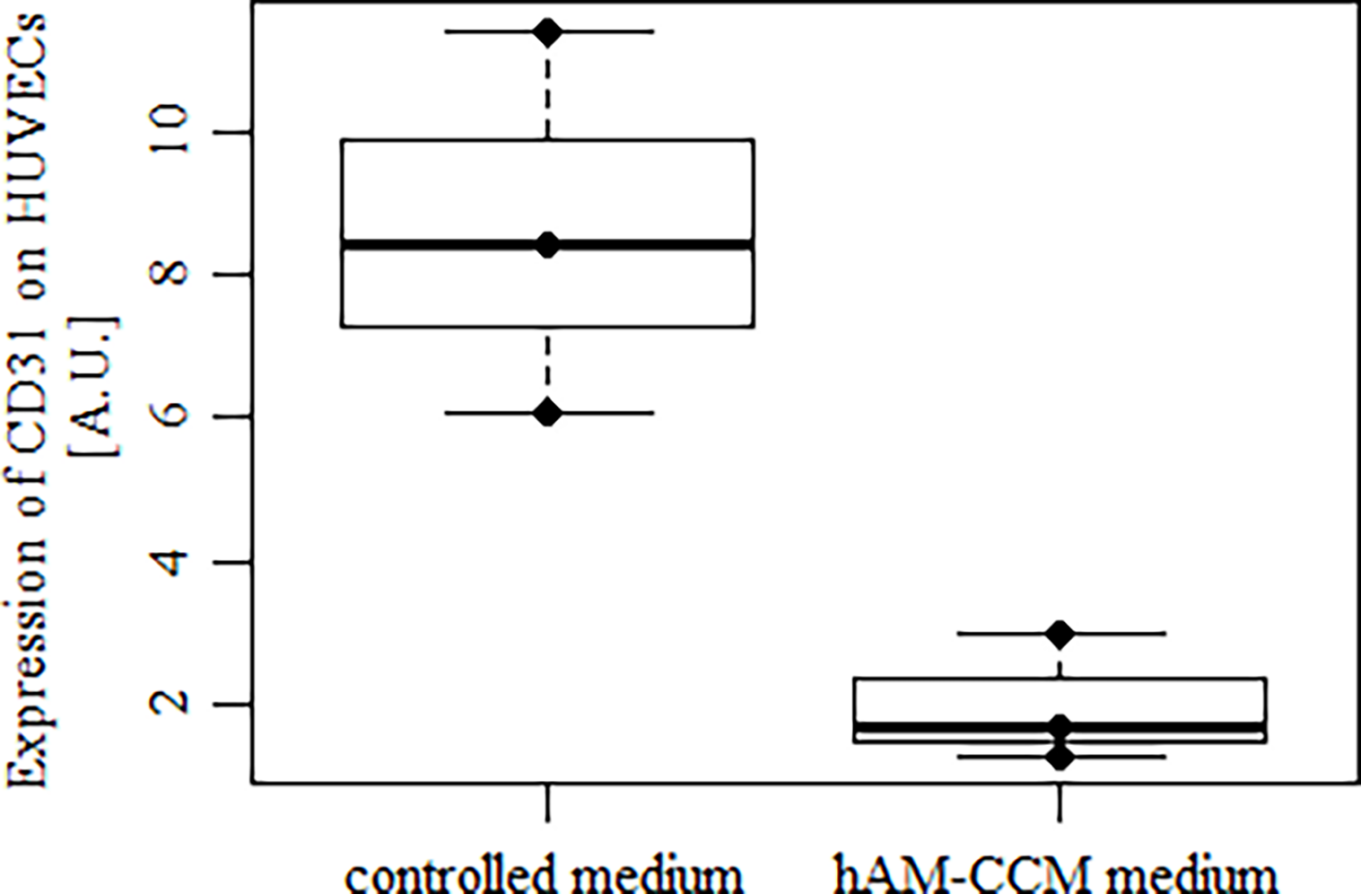
Effect of hAM CCM on HUVECs expression of cell adhesion molecule-1 (CD31) marker. Significantly lower expression is observed in presence of hAM CCM. Median values and interquartile ranges (P25, P75) are shown (n = 3, p < 0.05).

HUVECs marker protein CD31 is surface adherence antigen. When cells were intensely moving in presence of hAM CCM for 24 h the expression of cell adhesion molecule-1 (CD31) was significantly lower (4.3 fold) as compared to cells in control medium (Fig 4).

### Effect of hAM CCM on proliferation and migration of BM MNCs

**Fig 5:**
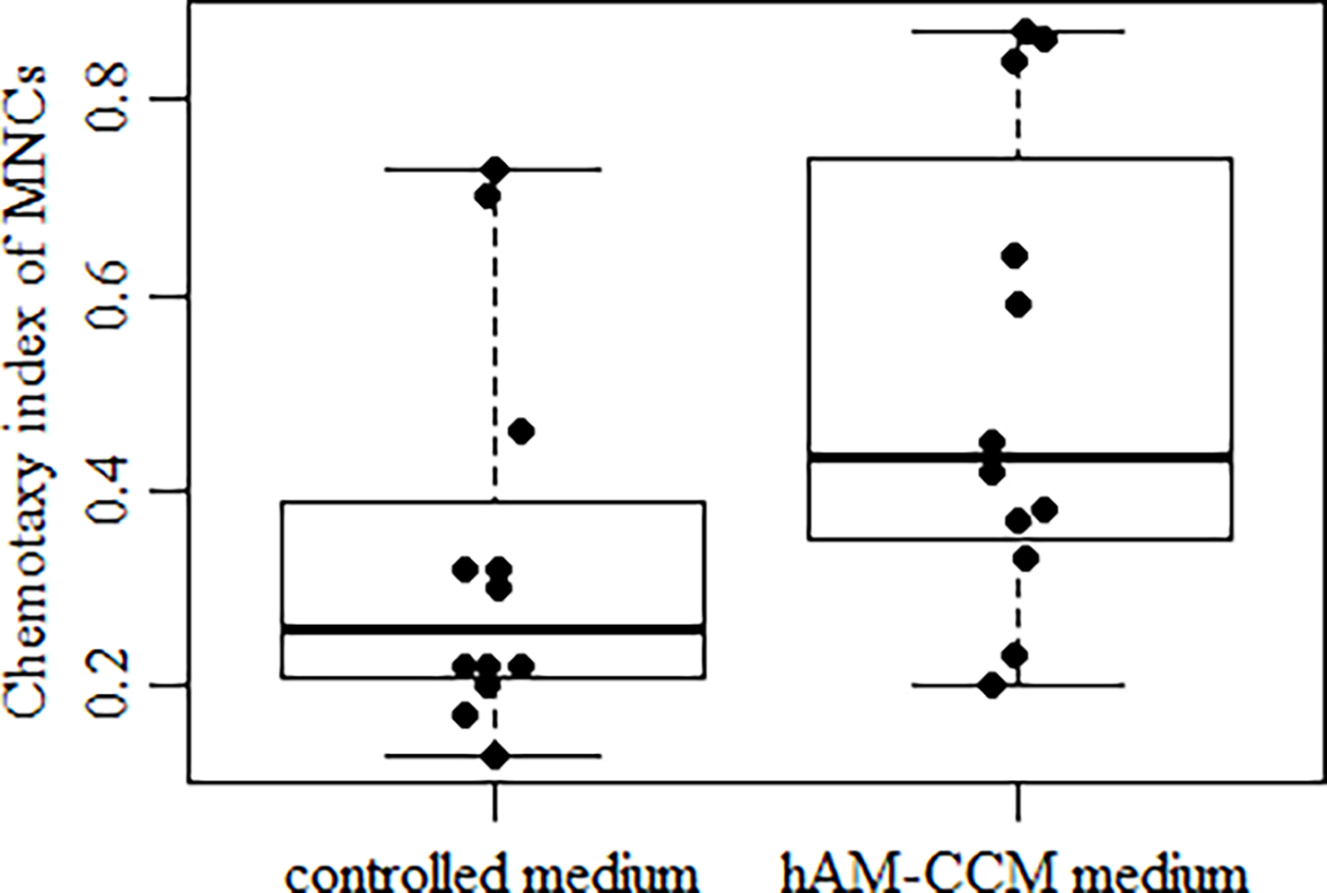
Effect of hAM CCM on chemotaxy indeks of BM MNCs. The chemotaxy index (CI) after 2.5 h at 37^°^C incubation time was calculated by dividing the number of cells in lower chamber by the number of cells added to the upper chamber counted at the start of the test. Median values and interquartile range (P25, P75) are shown (n = 12, p < 0.05).

Bone marrow MNCs were not proliferating in the presence of hAM CCM as compared with controls (zero proliferating cells difference) when assayed by Ki67 or PCNA antigen. The migration of bone marrow MNCs as tested in the Boyden chamber and evaluated by the with the chemotaxy index (CI) was significantly different for cells incubated with hAM CCM compared with controls. For cells migrating to the culture medium containing hAM CCM the CI was about 0.44 while for cells migrating to the control medium was about 0.27 and the difference was statistically significant (Fig 5). These results indicate that hAM CCM stimulates migration of BM MNCs.

### Effect of hAM CCM on proliferation and migration of Jurkat cells

**Fig 6:**
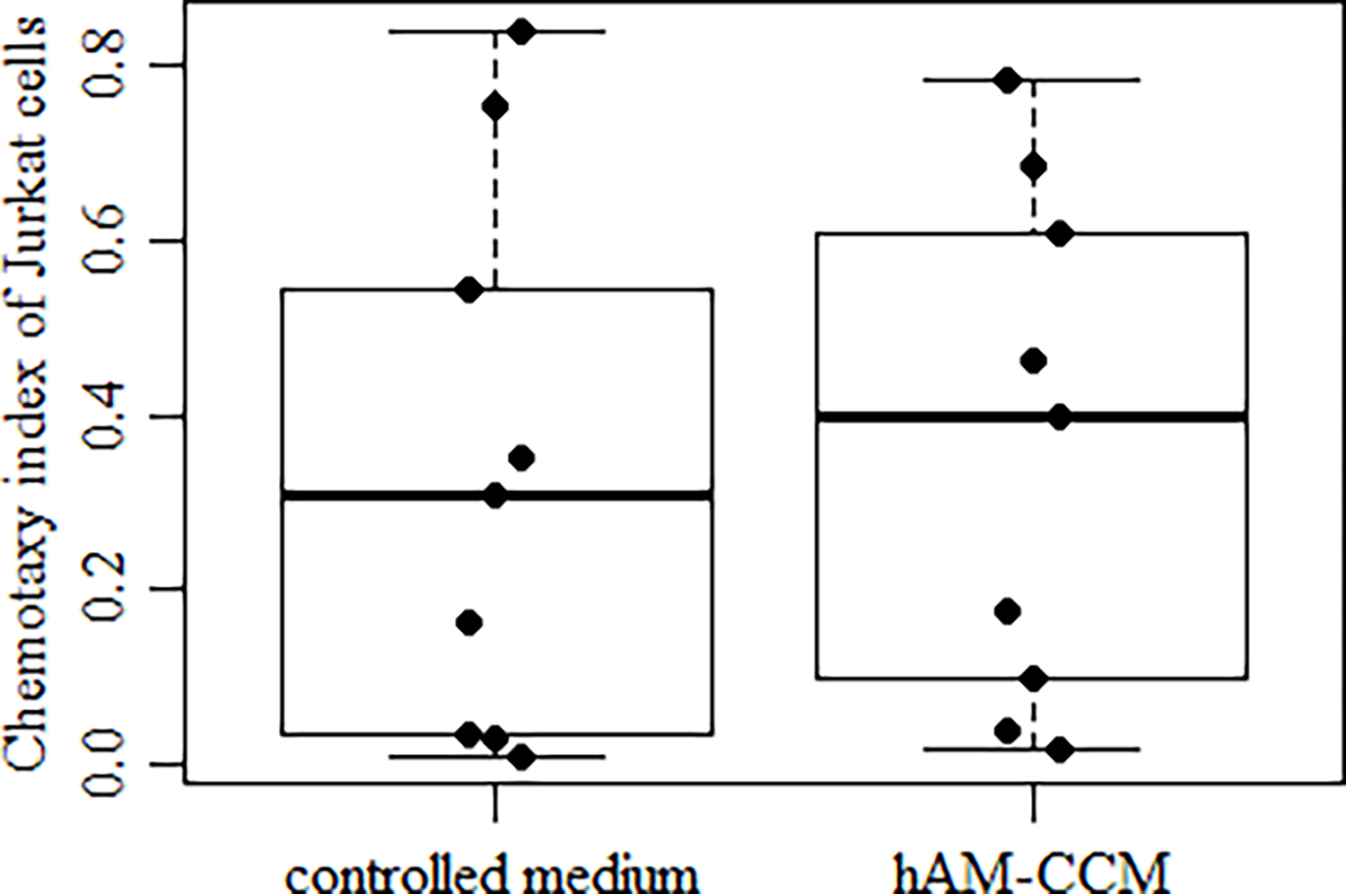
Effect of hAM CCM on migration of Jurkat cells. The cells were incubated in RPMI 1640 with 3% FBS and 2% human albumin medium for 4 h at 37^°^C. The chemotaxy index was calculated as in Fig 5. Median values and interquartile range (P25, P75) are shown (n = 9, p > 0.05).

Jurkat cell proliferation was assayed by measuring expression of Ki67 antigen and PCNA antigen. In the presence of hAM CCM, 7.9% (P25–P75: 7.1%–9.5%) of cells were Ki67 positive while in the control medium percentage was 7.7% (P25–P75: 6.3%–10.3%), with no statistically significant difference. Similarly the PCNA antigen assay yielded no significantly different results. Also migration of Jurkat cells in the Boyden chamber was not significantly modulated by hAM CCM (Fig 6). The median CI value with hAM CCM stimulation was 0.39 (P25–P75: 0.10–0.65); for non-stimulated cells, it was 0.31 (P25–P75: 0.03–0.65).

## Discussion

Amnion membrane fragments were found to release numerous growth factors into culture medium (hAM CCM). For practical purposes, especially for wound treatment, the possibility of using banked, sterile, ready-to-use hAM CCM may be of substanitial interest. Two functions of cells cultured in the presence of hAM CCM were assayed: proliferation and cell migration. The hAM CCM did not induce proliferation of the cells tested. However, the endothelial and bone marrow cells treated with hAM CCM had increased migration rates, which was not observed for human leukemia Jurkat cells. The faster migration of HUVECs and bone marrow cells to the hAM–CCM culture medium likely reflected the role of the amnion in blood vessel building and homing of hematopoietic cells helpful in a multifaceted therapy e.g. the chronic or toxic wound healing [10,11]. Cell mobility is an important function in angiogenesis angiogenic factors released from cryo-preserved AM preparations complementary to that observed here has been described [12]. The use of amniotic membrane in ophthalmic surgery has been shown to provide an alternative for corneal and conjunctival reconstruction in clinically challenging situations [6].

The Jurkat human leukemia cell line served as a control in observations of the induction of cell proliferation and mobility in normal cells. Neither parameter was significantly increased by hAM CCM in Jurkat cells despite the presence of TGF-β, an activator of proliferation process [13,14] in hAM CCM. These suggests more detailed tests with hAM CCM wound healing application in leukemia patients.

The observed IGF system is composed of three receptors (insulin receptor, IGF-1 receptor and IGF-2 receptor), three ligands (insulin, IGF-1, IGF-2), and six types of circulating IGF-binding proteins (IGFBP1–6) that modulate the availability and bioactivity of the IGFs [12,15]. The analyzed hAM CCM contained a growth factors from the IGF family (IGF-1, IGF-2), but failed to stimulate cell proliferation. This may be explained by comparing the levels of the IGF family members and of the IGFBP family proteins (IGFBP-1, -2, -3, -4, and -6). The identified IGF members were about five times less aboundant than all the IGFBPs combined. The IGF system has been reported to regulate tissue homeostasis and cellular metabolism by modulating microRNAs levels [16]. These regulatory effects may be expected in the amnion system with a high IGFBP secretion level.

Adhesion molecule CD31 (PECAM-1/EndoCAM) belong to the immunoglobulin supergene family expressed on endothelial cells, platelets, and some hematopoietic lineage cells [17,18]. We observed down-regulation of CD31 on mobility-activated HUVECs during migration induced by factors present in hAM CCM. However, in an experiment with CD31-deficient mice it was observed that the absence of the CD31 protein may be compensated by other adhesion molecules [19].

Several cytokines and ILs formerly thought to be specific for the hematopoietic system, notably granulocyte colony-stimulating factor (G-CSF), granulocyte–macrophage colony–stimulating factor (GM-CSF), and IL-3, -4, -6, and -8, can also affect the metabolism and functioning of endothelial cells [20–22]. Human recombinant GM-CSF as well as hrG-CSF have been shown to induce proliferation and mobility of HUVECs [22], however, the hAM CCM is a complex mixture of components in which some activities may be suppressed by other factors of opposite activity.

The HGF protein is present at high concentrations in our hAM CCM. HGF is known as a scatter factor combining mitogenetic and mobility activities [23,24]. An increased induction of erythroid burst-forming unit colonies from CD34^+^ cells of human BM MNCs was observed before in the presence of HGF and erythropoietin [25].

## Conclusions

It appears that the human amnion can be used as a source of chemotactic growth factors and erythropoiesis-stimulating HGF. In the current work, incubation of the human amnion led to the release of numerous factors into the culture medium (hAM CCM). The hAM CCM influenced HUVECs and bone marrow cells, which migrated more rapidly to the hAM–conditioned culture medium, suggesting some role in blood vessel building and homing of hematopoietic cells probably helpful in chronic or toxic wound healing. We also report the first identification of IGFBP family proteins secreted at high concentrations by the amnion, and discuss their possible function. We observed no response of Jurkat cells to hAM CCM, which indicates that application of the hAM medium should be possible also in leukemic patients. Overall, the results suggest that hAM can exert prolonged beneficial effects and that application of hAM CCM, possibly from a tissue bank, could aid wound healing.

## Supporting information

S1 Fig The example of real time proliferation assay result of stimulated and controlled HUVECs directly from the X-Celligence system. The difference between proliferation curves for cells in culture with presence of hAM CCM and in control medium was not significant for up to 100 h observation.
(TIF)

## References

[1] Grzywocz Z, Gawryluk B, Noszczyk B. Amnion membrane: structure, functions and applications in regenerative medicine. Adv Cell Biol 2012;39:637–652.

[2] Duan-Arnold Y, Gyurdieva A, Johnson A, Jacobstein DA, Danilkovitch A. Soluble factors released by endogenous viable cells enhance the antioxidant and chemoattractive activities of cryopreserved amniotic membrane. Adv in Wound Care 2015;4(6): 329–338. 10.1089/wound.2015.0637 PMC4440986 26029483

[3] Mermet I, Pottier N, Sainthillier JM, Malugani C, Cairey-Remonnay S, Maddens S, et al Use of amniotic membrane transplantation in the treatment of venous leg ulcers. Wound Rep Reg 2007;15:459–464. doi: 10.1111/j.1524-475X.2007.00252 10.1111/j.1524-475X.2007.00252.x 17650088

[4] Zhao B, Liu JQ, Zheng Z, Zhang J, Wang SY, Han SC, et al Human amniotic epithelial stem cells promote wound healing by facilitating migration and proliferation of keratinocytes via ERK, JNK and AKT signaling pathways. Cell Tissue Res 2016; 365:85–99. doi: 10.1007/s00441-016-2366-1 26888423 10.1007/s00441-016-2366-1

[5] Lo V, Pope E. Amniotic membrane use in dermatology. Int J Dermatol. 2009;48:935–40. doi: 10.1111/j.1365-4632.2009.04173.x 19702975 10.1111/j.1365-4632.2009.04173.x

[6] Gomes JA, Romano A, Santos MS, Dua HS. Amniotic membrane use in ophthalmology. Curr Opin Ophthalmol 2005;16:233–240. 16000896 10.1097/01.icu.0000172827.31985.3a

[7] Alio JL, Abad M, Scorsetti DH. Preparation, indications and results of human amniotic membrane transplantation for ocular surface disorders. Expert Rev Med Devices 2005; 2:153–160. doi: 10.1586/17434440.2.2.153 16293052 10.1586/17434440.2.2.153

[8] Sorsby A, Haythorne J, Reed H. Further experience with amniotic membrane grafts in caustic burns of the eye. Br J Ophtalmol 1947;31:409–418. 10.1136/bjo.31.7.409 PMC513860 18170361

[9] Grzywocz Z, Pius-Sadowska E, Klos P, Gryzik M, Wasilewska D, Aleksandrowicz B, et al Growth factors and their receptors derived from human amniotic cells in vitro. Folia Histochem Cytobiol 2014;52:163–170. doi: 10.5603/FHC.2014.0019 25308731 10.5603/FHC.2014.0019

[10] Koob TJ, Rennert R, Zabek N, Massee M, Lim JJ, Temenoff JS et al Biological properties of dehydrated human amnion/chorion composite graft: implications for chronic wound healing. Int Wound J. 2013;10:493–500. doi: 10.1111/iwj.12140 23902526 10.1111/iwj.12140 PMC4228928

[11] Steinritz D., Schmidt A., Balszuweit F., Thiermann H., Ibrahim M., Bölck B., Bloch W. Assessment of Endothelial Cell Migration After Exposure to Toxic Chemicals. J. Vis. Exp. (101), e52768, doi: 10.3791/52768 (2015). 26274775 10.3791/52768 PMC4544446

[12] Wolbank S, Hildner F, Redl H, van Griensven M, Gabriel C, Hennerbichler S. Impact of human amniotic membrane preparation on release of angiogenic factors. J Tissue Eng Regen Med 2009; 3: 651–654. doi: 10.1002/term.207 19701933 10.1002/term.207

[13] Mäkelä TP, Alitalo R, Paulsson Y, Westermark B, Heldin CH, Alitalo K. Regulation of platelet-derived growth factor gene expression by transforming growth factor beta and phorbol ester in human leukemia cell lines. Mol Cell Biol. 1987;7:3656–3662. 3479682 10.1128/mcb.7.10.3656-3662.1987 PMC368020

[14] Zloza A, Jagoda MC, Lyons GE Graves MC, Kohlhapp FJ, O’Sullivan JA, et al CD8 co-receptor promotes susceptibility of CD8+ T cells to transforming growth factor- (TGF-)-mediated suppression. Cancer Immunol Immunother. 2011;60:291–297. doi: 10.1007/s00262-010-0962-6 21193909 10.1007/s00262-010-0962-6 PMC4507403

[15] Kasprzak A, Kwasniewski W, Adamek A, Gozdzicka-Jozefiak A. Insulin-like growth factor (IGF) axis in cancerogenesis. Mutation Research/Reviews in Mutation Res 2017;772:78–104. 10.1016/j.mrrev.2016.08.007 28528692

[16] Jung HJ, YousinSuh Y. Regulationof IGF-1 signaling by microRNAs. Frontiers in Genetics 2015;5:Article 472 10.3389/fgene.2014.00472 PMC4292735 25628647

[17] Lertkiatmongkol P, Liao D, Mei H, Hu Y, Newman PJ. Endothelial functions of platelet/endothelial cell adhesion molecule-1 (CD31). Curr Opin Hematol. 2016;23:253–259. doi: 10.1097/MOH.0000000000000239 27055047 10.1097/MOH.0000000000000239 PMC4986701

[18] Park SY, Sorenson CM, Sheibani N. PECAM-1 Isoforms, eNOS, and Endoglin Axis in Regulation of Angiogenesis. Clin Sci (Lond). 2015; 129: 217–234. doi: 10.1042/CS20140714 25976664 10.1042/CS20140714 PMC4716661

[19] Duncan GS, Andrew DP, Takimoto H, Kaufman SA, Yoshida H, Spellberg J et al Genetic evidence for functional redundancy of platelet/endothelial cell adhesion molecule-1 (PECAM-1): CD31-deficient mice reveal PECAM-1-dependent and PECAM-1-independent functions. J.Immunology 1999; 162: 3022–3030. 10072554

[20] Colotta F, Bussolino F, Polentarutti N, Guglielmetti A, Sironi M, Bocchietto E, et al Differential expression of the common beta and specific alpha chains of the receptors for GM-CSF, IL-3, and IL-5 in endothelial cells. Exp Cell Res. 1993;206:311–317. doi: 10.1006/excr.1993.1151 7684696 10.1006/excr.1993.1151

[21] Kojima S, Tadenuma H, Inada Y, Saito Y. Enhancement of plasminogen activator activity in cultured endothelial cells by granulocyte colony-stimulating factor. J Cell Physiol. 1989;138:192–196. doi: 10.1002/jcp.1041380125 2463258 10.1002/jcp.1041380125

[22] Bussolino F, Ziche M, Wang JM, Alessi D, Morbidelli L, Cremona O et al In vitro and in vivo activation of endothelial cells by colony-stimulating factors. J Clin Invest. 1991;87:986–995. doi: 10.1172/JCI115107 1705569 10.1172/JCI115107 PMC329891

[23] Naldini L, Weidner M, Vigna E, Gaudino G, Bardelli A, Ponzetto C et al Scatter factor and hepatocyte growth factor are inditiguishable ligands for the Met receptor. EMBO J 1991;10:2867–78. 1655405 10.1002/j.1460-2075.1991.tb07836.x PMC452997

[24] Weidner K, Arakaki N, Hartmann G, Vandekerchove J, Weingart S, Rieder H et al Evidence for the identiti of human scatter factor and hepatocyte growth factor. Proc Natl Acad Sci USA 1991;88:7001–5 1831266 10.1073/pnas.88.16.7001 PMC52221

[25] Galimi F, Bagnara GP, Bonsi L, Cottone E, Follenzi A, Simeone A, Comoglio PM. Hepatocyte growth factor induces proliferation and differentiation of multipotent and erythroid hemopoietic progenitors. J Cell Biol 1994;127:1743–54 7528222 10.1083/jcb.127.6.1743 PMC2120271

