# Supplementary figures and images for: Response of human normal and leukemia cells to factors released by amnion fragments in vitro
Source: PLoS One. 2018 Mar 29;13(3):e0195035. doi: 10.1371/journal.pone.0195035 (PMC5875856; doi:10.1371/journal.pone.0195035)

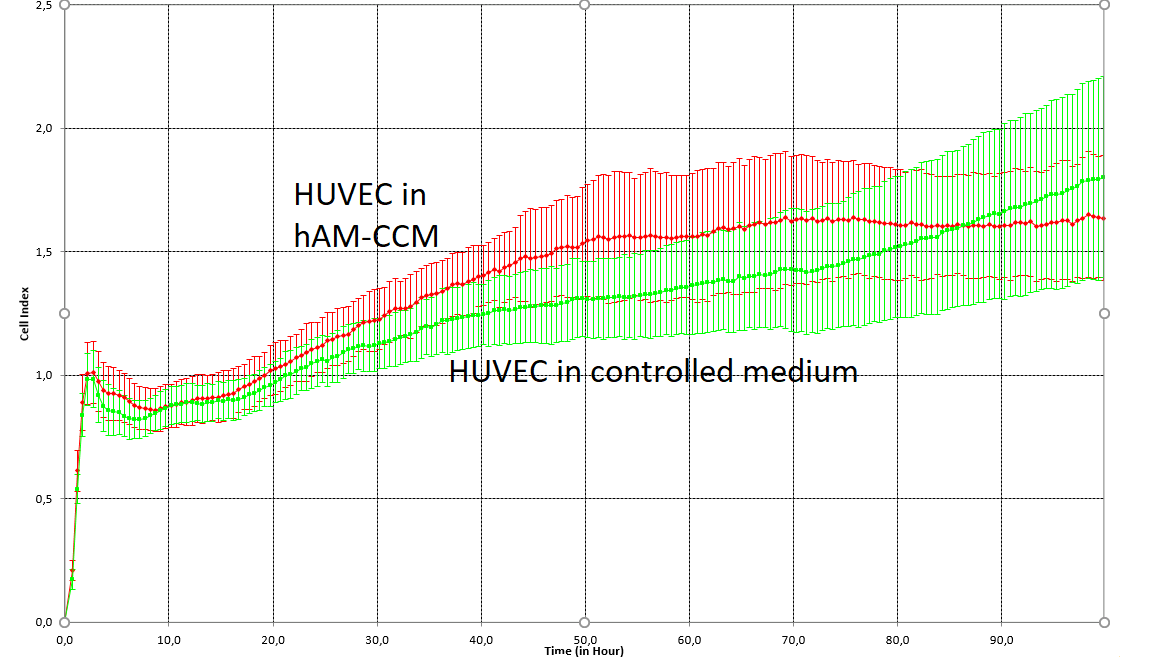

Supplement: S1 Fig — The difference between proliferation curves for cells in culture with presence of hAM CCM and in control medium was not significant for up to 100 h observation. (TIF) [file pone.0195035.s001.tif]
